# Designs of Antigen Structure and Composition for Improved Protein-Based Vaccine Efficacy

**DOI:** 10.3389/fimmu.2020.00283

**Published:** 2020-02-24

**Authors:** Kyle Saylor, Frank Gillam, Taylor Lohneis, Chenming Zhang

**Affiliations:** ^1^Department of Biological Systems Engineering, Virginia Tech, Blacksburg, VA, United States; ^2^Locus Biosciences, Morrisville, NC, United States; ^3^BioPharmaceutical Technology Department, GlaxoSmithKline, Rockville, MD, United States

**Keywords:** vaccine, immunity, antigen, epitope, modification, vaccine composition, vaccine structure

## Abstract

Today, vaccinologists have come to understand that the hallmark of any protective immune response is the antigen. However, it is not the whole antigen that dictates the immune response, but rather the various parts comprising the whole that are capable of influencing immunogenicity. Protein-based antigens hold particular importance within this structural approach to understanding immunity because, though different molecules can serve as antigens, only proteins are capable of inducing both cellular and humoral immunity. This fact, coupled with the versatility and customizability of proteins when considering vaccine design applications, makes protein-based vaccines (PBVs) one of today's most promising technologies for artificially inducing immunity. In this review, we follow the development of PBV technologies through time and discuss the antigen-specific receptors that are most critical to any immune response: pattern recognition receptors, B cell receptors, and T cell receptors. Knowledge of these receptors and their ligands has become exceptionally valuable in the field of vaccinology, where today it is possible to make drastic modifications to PBV structure, from primary to quaternary, in order to promote recognition of target epitopes, potentiate vaccine immunogenicity, and prevent antigen-associated complications. Additionally, these modifications have made it possible to control immune responses by modulating stability and targeting PBV to key immune cells. Consequently, careful consideration should be given to protein structure when designing PBVs in the future in order to potentiate PBV efficacy.

## Introduction

Physicians have been aware of the effects of vaccination for over a millennium, though they had little understanding of the mechanisms through which immunity was achieved until the early nineteenth century. From the simple and dangerous live-pathogen variolation employed in ancient and medieval times to the recombinant protein and DNA vaccines we use today, vaccine development has followed a path of improving efficacy and safety due to an ever-increasing understanding of these mechanisms. The hallmarks of this understanding have been that an adaptive immune response cannot take place without an antigen and that the most effective antigens tend to be proteins. Ultimately, these two facts have led the field of vaccinology to focus on the research and development of protein-based vaccine (PBVs). As such, we now have a dense literature pool that can be used to recapitulate past and present PBV design concepts and illuminate fundamental structural vaccinology principles in PBV design.

Bacterial toxin vaccines were the first PBVs to be developed. They originally consisted of antitoxin isolated from animals inoculated with small quantities of unmodified toxin, but later it was discovered that active immunization could be safely achieved if toxin was either (1) co-administered with a sufficient amount of antitoxin (partial neutralization) or (2) treated chemically or thermally prior to vaccine administration (denaturation). Chemically inactivated toxin, dubbed toxoid, would go on to garner widespread celebrity due to its success in World War II and eventually become the primary means for immunization against Diphtheria and Tetanus (1). The development of these toxoid vaccines, along with the gradual acceptance of the side chain theory, the establishment of immunological proteomics, and the application of genetic engineering technologies, paved the way for the emergence of the PBVs we know today (2).

Recombinant vaccines, as their name suggests, are PBVs that are produced using recombinant DNA technology. The first recombinant PBV was produced in yeast in 1986 and targeted the surface antigen of the Hepatitis B virus (HBV) (3). Though it was one of the first recombinant proteins to be approved for use in humans, its discovery was largely overlooked due to perceived limitation in likely impact coupled with the fact that a serum-derived vaccine for HBV already existed (3). However, the success of this virus-like particle-based (VLP) PBV spurred an increased interest in whether the same recombinant strategy could be implemented with other viruses. This idea flourished over time, and today recombinant vaccine approaches have been attempted for nearly every viral structural protein identified. Results from this research have been considerable, with five VLP-based PBVs having been commercialized and dozens having reached clinical trials (4). It is important to note, however, that recombinant PBVs are not limited to VLP formulations, as many bacterial pathogen-derived and tumor-associated antigen-derived recombinant PBVs have also been developed (5, 6).

Conjugate vaccines, which consist of carrier protein—subunit complex, were conceived in the late 1980's in order to address a growing need for more effective vaccines against encapsulated bacterial pathogens from the *Neisseria, Streptococcus, Staphylococcus, Haemophilus*, and *Pseudomonas* generas. Before their discovery, vaccine formulations targeting these pathogens singularly consisted of polysaccharide (typically the exposed glycan from encapsulated bacterial surfaces). Although these polysaccharide vaccines were shown to elicit the production of protective antibodies, they proved to be tremendously ineffective at conferring protection in young and immunocompromised individuals and largely failed to elicit immunological memory (7). The limited success of the first subunit polysaccharide vaccines was eventually concomitant to the discovery that polysaccharide vaccines are unable to recruit the assistance of T helper cells and thus rely on T cell-independent activation alone (8). Protein-based, subunit vaccines, in contrast, were found to have all the components necessary to initiate T cell-dependent activation of B cells, a process characterized by a more robust immune response, affinity maturation, and immunological memory (9).

**Figure 1:**
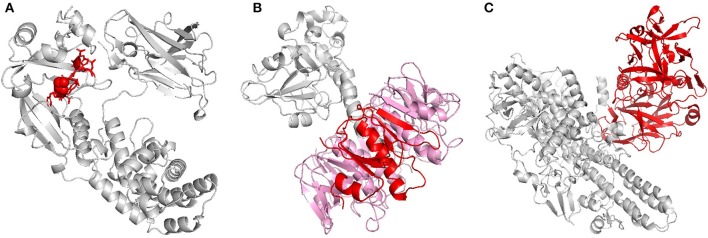
Recombinant toxins. **(A)** Diphtheria toxin (DT), when replacing glycine with glutamic acid at position 52, loses its toxicity without affecting its antigenicity. The highlighted residues (red) indicate the exact residue (sphere) and area (licorice) where this substitution would occur on monomeric DT. **(B)** Cholera toxin (CT) is composed of six subunits; one A subunit and five B subunits. B subunit (monomer in red, remaining subunits in pink), which lacks the toxicity of its partner A subunit, has proven to be extremely immunogenic and is used as a carrier protein and adjuvant. B subunit of heat-labile enterotoxin, which shares much of the same homology as B subunit of cholera toxin, has been similarly investigated (12). **(C)** Tetanus toxin (TT) is comprised of two chains, a light chain and a heavy chain, of which the light chain is responsible for the protein's toxicity. In the past, proteolytic digestion of TT with papain yielded two fragments, a light chain-containing, toxic B fragment and a non-toxic C fragment (red). Vaccination with the non-toxic C fragment was found to be protective against lethal toxin dose in a mouse model, and today PBVs comprised of recombinant C fragment are being investigated as a potential replacement for TT vaccines. Botulinum toxin, which shares much of the same homology as tetanus toxin, has been similarly investigated (13). The 3D protein structures for DT, CT, and TT used in this image were rendered in PyMOL 2.3.0 and accessed via the Protein Data Bank (14–18).

Toxoids have traditionally been used as carrier proteins in conjugate PBV formulations because of their excellent immunogenicity, availability, and simplicity (10). Many of the conjugate PBVs being developed today, however, use recombinantly produced carrier proteins that have been specifically designed to maximize efficacy while simultaneously maintaining a good safety profile (11). The first carrier protein of this type, cross-reactive material 197 (CRM197), was discovered upon the random, mutagenic conversion of glutamic acid to glycine at position 52 of diphtheria toxin (DT, Figure 1A). Though distal to the ADP-ribosyltransferase active site found on the A subunit of DT, this single point mutation on the B subunit was able to completely eliminate DT's toxicity without negatively impacting its ability to stimulate the immune system (19–21). The discovery of CRM197 ultimately led to the realization that the inherent toxicity of the antigens typically employed in conjugate PBV formulations could be modulated using precise structural modifications as opposed to broad-based chemical and thermal denaturation. Thus, the idea of structure-based vaccinology was born and a growing trend in research involving “designer vaccines” began. Since its conception, this concept has been applied to a plethora of pathogenic determinants, specifically toxins. It was observed that the use of cholera toxin B subunit (CTB) in PBV formulations, as opposed to complete toxin, could lead to improved safety profiles with little-to-no decline in overall immunogenicity (Figure 1B). The improved safety was attributed to the missing A1 domain, the portion of cholera toxin responsible for intracellular activity that leads to disease symptoms (22). A similar discovery was made for tetanus toxin when it was revealed that the heavy chain C fragment (TTc), when used as an immunogen, could confer protection upon toxin challenge in a mouse model without eliciting the neurotoxic effects of its parent protein (Figure 1C) (23). Unfortunately, the use of TTc in modern vaccines may be discouraged by its capacity to bind neurons, though researchers have undertaken structural and conformational approaches to the modulation of this interaction (23, 24). Similar methods to those outlined here have also be employed with other toxins, such as heat-liable enterotoxin (a close relative of cholera toxin) and botulinum toxin (a close relative of tetanus toxin) (12, 25).

**Figure 2:**
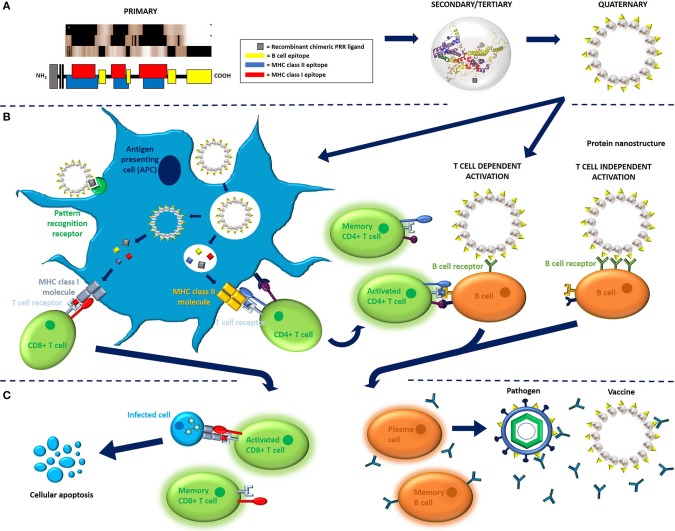
Immunological mechanisms of recombinant, protein-based vaccination. **(A)** PBV structure, as illustrated here for the model protein hepatitis B core antigen (HBcAg, 183 aa long, non-truncated form, Accession number P03146), is ultimately determined by primary sequence. Vaccine can comprise monomeric antigen (i.e., toxoid protein) or multimeric antigen (i.e., virus-like particles), though multimeric antigen is used for demonstration purposes here. T cell and B cell antigenic determinants can be identified in primary sequence using various *in vitro* and *in silico* methods. The linear MHC epitopes illustrated here were predicted using Epitope Analysis Resources on the Immune Epitope Database (IEDB) website. More specifically, MHC epitopes were predicted for HLA-A*02:01 (class I molecules) and all heterodimer combinations of DQB1*02:01 (class II molecules) using IEDB recommended methods. Linear B cell epitopes, on the other hand, were assigned using frequency analysis results from the IEDB website (26). PBV structures are color coded to represent epitope content. **(B)** Cell processing and activation in response to PBV is generally orchestrated by antigen presenting cells, of which the most important are dendritic cells. APCs sample their environment via endocytosis, specifically via receptor-mediated endocytosis when antigen presents glycan and/or protein pathogen-associated molecular patterns such as high mannose glycans or bacterial flagellin. Depending on the structural and compositional characteristics of the antigen, APCs will either process antigen via MHC class II pathway or MHC class I pathway using a mechanism known as cross-presentation, respectively, resulting in activation of either CD4+ (helper) or CD8+ (cytotoxic) T cell response. CD4+ T cell activation requires co-activating signals and results in the proliferation of effector and memory CD4+ T cell pools. Effector CD4+ T cells go on to assist with the activation of B cells (T cell dependent activation) and provide survival signals to activated CD8+ T cells, whereas CD8+ T cells have immediate effector functionality. B cells can also undergo T cell independent activation when antigen cross-links multiple BCRs on B cells surface (TI-2 activation) or co-signals via PRR (TI-1 activation) (27, 28). **(C)** Activation results in the proliferation of memory and effector cytotoxic T cell and B cell pools. Memory CD8+ cells remain dormant until they encounter cells presenting MHC class I molecules loaded with cognate epitope, upon which they begin mounting an effector response. Effector CD8+ T cells go on to instruct apoptosis in cells presenting MHC class I molecules loaded with cognate epitope. B cells activated via T cell independent pathway generally proliferate into short-lived plasmablasts that express low affinity IgM antibodies (not shown). B cells activated via T cell dependent pathway, on the other hand, result in the proliferation of memory B cells and long-lived plasma cells expressing high-affinity IgA, IgE, or IgG antibodies. Antibodies secreted by plasms cells (and plasmablasts) go on to bind vaccine and pathogen and initiate antibody effector functions. Memory B cells remain dormant until they encounter antigen presenting cognate epitope, upon which they rapidly proliferate and clones either class switch to become antibody secreting plasma cells or re-enter germinal centers and restart affinity maturation processes (27, 28). The 3D protein structure for HBcAg used in this image was rendered in PyMOL 2.3.0 and accessed via the Protein Data Bank (14, 18, 29).

**Figure 3:**
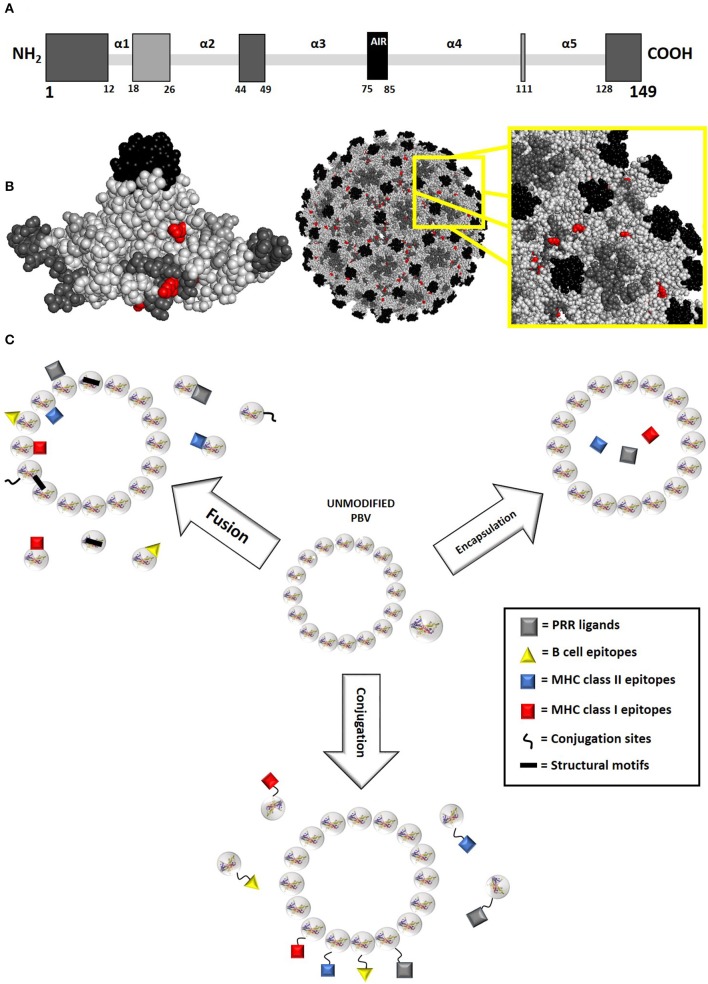
PBV modification principles. **(A)** Potential fusion modifications sites for the model protein hepatitis B core antigen (HBcAg, 149 aa long, truncated form of AN P03146 used for vaccine purposes) as represented by primary structure. Residues are color-coded in gray scale, with darker residues indicating more exposed insertion locations. Polypeptide termini and random coil loop regions are primary targets for PBV fusion modification, as they generally have the least effect on protein structure. Within these considerations, surface regions that do not participate in intra- and intermolecular interactions are preferred. Specifically, most HBcAg fusion PBVs presenting foreign B cell epitopes have been modified within the α3α4 loop (AIR between amino acids 75 and 85), though modifications are also routinely made at the C and N termini. A different approach to insertion site selection should be taken when creating fusion PBVs targeting T cell immune responses, as antibody response to epitope becomes detrimental. Toward this goal, inserting epitopes within loop regions that are less exposed and less likely to negatively influence protein stability is optimal (31, 32). **(B)** Potential fusion modification and conjugation sites for truncated HBcAg model protein as represented by higher order folded and assembled structure. Residues are color-coded in gray scale, with darker residues indicating more exposed insertion locations. Natural conjugation sites (lysine and cysteine) have also been highlighted in red. **(C)** PBV modifications are generally orchestrated via fusion, conjugation, or encapsulation. Each type of modification occurs at a different level of protein structure, with fusion inserts occurring within primary structure, conjugated inserts occurring within secondary/tertiary structure, and encapsulated inserts occurring within the quaternary structure of proteins that form enclosed, organized matrixes. As such, both monomeric and multimeric protein can accommodate fusion and conjugation modifications, whereas only multimeric protein can accommodate encapsulation. The 3D protein structure for HBcAg used in this image was rendered in PyMOL 2.3.0 and accessed via the Protein Data Bank (14, 18, 29).

Today, interest in “designer vaccines” has been increasingly fueled by advancements in our understanding of the mechanisms behind innate and adaptive immunity, specifically the role of antigen composition in PBV immunogenicity (Figures 2A–C). X-ray crystallography, genetic sequencing, epitope prediction algorithms, and *in vivo* studies of adjuvant properties have all lead to a better understanding of why some proteins are simply more immunogenic than others. Ultimately, differences in protein structure can result in different capacities for antigen to interact with cells and receptors that are key to triggering an immune response (30). Knowledge of this phenomenon has led to a situation where vaccines are no longer conceptualized based on whole antigen, but rather on immune receptor epitopes/ligands and propensity of an antigen (based on structural motifs) for uptake by antigen presenting cells (APCs). As such, many of today's PBVs are engineered using structural vaccinology principles and rationally target APCs and the three receptor groups key to any adaptive immune response; the pattern recognition receptors (PRRs), the B cell receptors (BCRs), and the T cell receptors (Figures 3A–C).

## Targeting Pattern Recognition Receptors

### PBVs, PRRs, and the Innate Immune System

PRRs are specific for highly conserved molecular signals indicating the presence of cell damage and/or pathogens. They activate the innate arm of the immune system, resulting in the production of pro-inflammatory cytokines and chemokines. This activation assists in the maturation of the adaptive immune response and can be critical to the success or failure of a PBV. There are a myriad of PRRs that have been discovered that recognize either pathogen associated molecular patterns (PAMPs) or damage associated molecular patterns (DAMPs) (33). Generally, it is the responsibility of the adjuvant in vaccine formulations to activate the innate immune system. To this effect, researchers usually co-administer free PRR ligand, such as Toll-like receptors (TLRs, intracellular and extracellular membrane-bound PRRs), NOD-like receptors (NLRs, intracellular cytosolic PRRs), C-type lectin receptors (CLRs, extracellular membrane-bound PRRs), and/or RIG-I-like receptor (RLRs, intracellular cytosolic PRRs) agonists with the target antigen when they want to potentiate PBV immunogenicity (34). This potentiation is crucial to PBV success, as choice of adjuvant has a profound influence on both the magnitude and type of immune response the vaccine ultimately elicits (for more on this, please refer to the excellent reviews of Del Giudice et al. and Bonam et al.) (35, 36). It is also possible to rationally incorporate PRR ligands with PBVs using various techniques, an approach that imparts a distinct advantage over simple co-formulation in that the immunogen maintains proximity to the antigen.

### Rational Incorporation of PTMs as PAMPs

One strategy for the incorporation of PRR agonist is the manipulation of post translational modifications (PTMs) through the selection of the expression host (37). Since PTM types and sites vary widely between species, they are believed to direct the innate immune response against antigen (38). The PTM we know most about when concerning vaccine immunogenicity is glycosylation. With the exception of *E. coli*, all expression hosts that are commonly used for recombinant PBV production incorporate glycans on protein surfaces (39). These glycans can serve as ligands for many PRRs, specifically those of the CLR family (40). In fact, glycans have long been known to elicit immune responses to recombinant therapeutic proteins (41–43), and many of the potent adjuvants co-formulated with today's PBVs are polysaccharides (44–48). In one instance, glycosylation patterns were even shown to potentiate immune response to subunit vaccine (49). A thorough literature search, however, indicated that the modulation of PBV immunogenicity via the rational selection of the expression host has never been directly assessed. Considering that PTMs are incorporated *in vivo* on specific, surface-exposed amino acids found on most proteins, it should be possible to exploit expression host glycosylation patterns when designing PBVs. Likewise, the potentiation of post-translational modification through recombinant addition of these residues within protein primary structure where modification is likely to occur might prove to be an effective means of improving PBV immunogenicity. Detrimentally, however, PRR ligands also have the capacity to act as BCR and TCR epitopes (also known as antigenic determinants) and stereometrically crowd antigen surface. This can result in masked immunogenicity of and/or immune responses being redirected away from important epitopes contained within PBVs. A perfect example of this phenomenon was reported by Ansari et al. when they observed improved *in vitro* viral neutralization and *in vivo* epitope-specific antibody response to glycoprotein 5 (GP5) of the porcine reproductive and respiratory syndrome virus (PRRSV) when key N-linked glycosylation sites were eliminated from the immunodominant, N-terminal ectodomain (50).

### Facilitating PAMP Proximity to PBV via Fusion

Another way to engineer PBVs that can directly engage a wide variety of PRRs is to covalently fuse PRR agonists to the PBV. This can be accomplished during protein synthesis via the inclusion of the gene for a protein adjuvant within the open reading frame of the target antigen (chimeric approach) or post synthesis by using a number of biochemical techniques (conjugate approach). Mechanistically, fusion of protein-based PRR ligands to PBVs ensures proximity of adjuvant to antigen, thus increasing the likelihood of antigen-adjuvant co-delivery to key immune cells. Ultimately, this approach to adjuvanting is intended to improve immune response profile and intensity while simultaneously minimizing off-target effects (51). It is important to note, however, that chimeric and conjugate modification of antigen with PRR ligand can have detrimental impacts similar to those mentioned previously for natural PRR ligand incorporation. For example, one study was able to show that the chemical conjugation of imidazoquinoline compound 3M-012, a TLR7/8 agonist, to HIV envelope glycoprotein gp120 results in improved *in vitro* expression of IFNα by peripheral dendritic cells (DCs) while simultaneously abrogating the binding of critical, broadly neutralizing antibodies (52).

#### Chemical Conjugation of PAMP to PBV

Chemical conjugation of PRR to PBV surface has been explored by many research groups. Chang et al. attached flagellin to the surface of ovalbumin (OVA) nanoparticles (NPs) and observed improved TLR5 activation in HeLa cells when compared with OVA NPs without adjuvant. However, there was no statistical difference when comparing OVA NPs that were conjugated to flagellin with those that were co-administered. These results indicated that conjugating adjuvant to antigen is a viable means of activating the innate immune system, but failed to implicate an effect of adjuvant proximity to antigen on PRR signaling (53). Alternatively, Kastenmüller et al. found that conjugation of TLR7/8 agonist to OVA improved DC uptake of antigen and subsequent innate immune activation when compared to co-administered antigen and adjuvant (54). A similar study targeted TLR7 by chemically conjugating the adenine-based adjuvant SA-26E to recombinantly expressed group 2 allergen from the house dust mite. Results indicated that conjugation not only improved innate immune response when compared to co-administered antigen and adjuvant, but also that conjugation was capable of redirecting immune response away from the Th2 cell subtype associated with hypersensitivity (55). Tighe et al. observed comparable results when conjugation, but not coadministration, of CpG oligonucleotide (CpG ODN, a TLR9 agonist) with the major short ragweed allergen Amb a 1 led to polarization of Th1 response in mice and higher IgG antibody titers in rabbits and monkeys (56). Schulke et al. observed enhanced secretion of all evaluated pro-inflammatory cytokines without bias toward the activation of any one T cell subtype when monophosphoryl lipid A (MPLA) was chemically conjugated to OVA and used to stimulate DC/T cell co-cultures (57). In one example of a PBV targeting a tumor associated antigen (TAA), McKee et al. observed increased activation of gp33-specific cells and prophylactic protection against tumor challenge in a mouse model when they conjugated α-galactosylceramide (a CD1d agonist) to a chimeric VLP consisting of gp33 MHC class I epitope and rabbit hemorrhagic disease virus protein (58). Finally, results from two additional studies indicate that adjuvant-mediated activation of the innate immune system is variable between species and that LPS and MPLA, two PRR ligands that signal through the same TLR4 receptor, has differing capacities to activate the innate immune system *in vitro* (59, 60). Together, these results indicate that PRR conjugation is a viable means of potentiating innate immune system activation, though it appears that there is still more to understand when considering the exact nature of the resulting immune response.

#### Genetic Fusion of PAMP to PBV

One example of recombinant fusion of PRR with PBVs are flagellin-fused antigens. These vaccines have intrinsic self-adjuvanting properties through their activation of TLR5 and, if the PBV is able to survive endocytosis, the NLRs NLRC4 and NAIP5 (61, 62). In one study assessing this approach, an 11.9-fold increase in hemagglutination inhibition assay titers was observed when a flagellin-fused hemagglutinin vaccine was compared with commercial influenza PBVs (63). Another promising study reported improved antibody titers (~60% increase), improved viral neutralization titers (~3-fold increase), and improved Th1 cytokine profile (~2-fold increase in IFNγ and TNFα expression) when comparing the effectiveness of flagellin-modified and wild-type porcine circovirus type 2 Cap protein (64). Additionally, many other studies evaluating the effectiveness of recombinantly fusing flagellin to PBV have shown promising results, with the majority reporting improved overall immunogenicity and/or survival when compared to antigen administered alone (65–71). Unsurprisingly, results like these have propelled multiple flagellin-fused PBVs targeting influenza to clinical trials (72–75). None of these candidates achieved commercialization, however, possibly due to the cytokine storm (a phenomenon that occurs when the immune system uncontrollably releases proinflammatory signals) observed upon vaccine administration in clinical trials (74). Nonetheless, structurally modified flagellin is still being investigated as a potential PRR fusion partner for antigen within recombinant PBV formulations (76, 77).

Another example of the PBV-PRR fusion approach to vaccination are recombinant and synthetic lipoprotein-based vaccines (LPBVs). These have become a popular research topic due to their ability to self-adjuvant via TLR2/1–6 heterodimers (78). LPBVs consist of a polypeptide sequence with a PRR-activating, N-terminal diacyl or triacyl lipid attachment (79). Incorporation of target epitopes using chimeric and conjugate approaches have been demonstrated in LPBVs targeting tuberculosis (TB), human papilloma virus, hepatitis C virus, influenza A virus, human immunodeficiency virus (HIV), and cancer (80–85). Generally, augmentation of immunogenicity has been reported in these studies, though it is important to note that prophylactic efficacy of the TB vaccine and therapeutic efficacy of the HIV vaccine was not observed (81, 84). Expression host may be an important factor in LPBV production as fatty acid incorporation *in vivo* not only varies among species but also results in a mixture of different lipoprotein structures that may have varying immunomodulating properties (86). This principle, however, has only been demonstrated when comparing the immunogenicity of naturally and synthetically produced LPBVs (87).

### Facilitating PAMP Proximity to PBV via Encapsulation

PRR agonists can also be encapsulated by antigen when working with proteins that self-assemble into organized matrixes, as has been demonstrated by the “packaging” of nucleic acids and proteins within various VLPs (88–93). Post-expression encapsulation can be achieved via simple diffusion through vaccine matrix pores when working with nucleic acids, though assembly and disassembly cycling may be necessary to encapsulate larger particles, such as proteins. The efficiency of this process, at least when considering nucleic acids, is attributed to electrostatic interactions between PBV interior and PRR agonists (94). The natural encapsulation of ssRNA upon expression of recombinant, RNA-containing bacteriophage VLPs in *E. coli* has been observed, illuminating another mode in which choice of expression host can influence antigen composition (95). Encapsulation has been shown to improve the half-life of nucleic acids (over 9-fold in some instances), most likely due to the reduced access of endonucleases mediated by vaccine matrix (96, 97). Additionally, as vaccine matrix payload won't be accessible to PRRs until after cellular uptake of vaccine, this approach ensures that encapsulated PRR agonists are more effectively delivered to endosomal and/or cytosolic PRRs (4). This is especially important for nucleic acid PRR agonists, as all PRRs recognizing nucleic acids are intracellular (98). Finally, encapsulation of PRR agonist may also prove advantageous in the design of PBVs targeting TAAs, as the efficacy of these vaccines is generally reliant on the activation of cytotoxic lymphocytes (99). This may be the reason why the CpG-ODN loaded MelQbG10 VLP based cancer vaccine (Qbeta VLP covalently linked to TAA MART-1_16−35_) developed by Speiser et al. was able to successfully reach Phase IIa in clinical trials (100).

## Targeting B Cell Receptors

### PBVs and the B Cell

BCRs (membrane bound immunoglobulin-CD79 protein complexes) and the B cell maturation process are used by the adaptive immune system to identify and neutralize linear and conformational epitopes exposed on the surface of antigens. Their activation eventually results in the proliferation of plasma cells and the subsequent secretion of antibodies that are highly specific for their target epitope. This, in turn, confers protection through various mechanisms that eventually result in antigen clearance and further stimulation of both the adaptive and innate immune system (27). Targeting the BCR in a vaccination strategy can therefore greatly impact the efficacy of a vaccine, especially if antibody-mediated neutralization or sequestration is required.

### Mechanisms Behind BCR Recognition of PBV

Many structures can serve as BCR epitopes in PBV designs, though there are some limitations associated with BCR complimentary determining region (CDR, the portion of an immunoglobulin or TCR that interacts with antigen) binding (101). To start, initial BCR CDR binding is largely beyond the control of PBV design. This is because BCR CDR structure, and therefore binding, is randomly determined in pro-B cells via variable, diversity, and joining (VDJ) gene recombination (102). PBV design can influence BCR CDR structure during subsequent, somatic hypermutation rearrangements through many different mechanisms, though efficient control over these rearrangements is difficult to achieve (103). These mechanisms are mostly epitope-specific and include epitope localization within antigen, competition between epitopes, epitope shape, and epitope size. BCR epitope localization is an important consideration in PBV design because exposed regions on antigen surface will always have the highest probability of becoming antibody immunodominant regions (AIRs) due to increased BCR access (104). Epitope competition refers to situations in which a PBV has multiple AIRs that compete with each other for BCR recognition. This results in the generation of multiple, polyclonal memory B cell pools that compete with each other for PBV upon boosting, an effect that can be detrimental when attempting to target or avoid specific AIRs (105–107). Epitope shape refers to the inherent ability of a conformational epitope to fit within the CDR of a BCR. The sequence of a linear epitope similarly influences BCR recognition and can be included in this category. Prediction software has been developed for the purpose of recognizing both conformational and linear BCR epitopes, indicating that receptors do preferentially interact with epitopes displaying certain quantifiable properties (i.e., patterns in sequence) (108). However, the utility of these predictions when considering PBV design is limited due to small and inconsistent datasets, difficulties predicting antigen 3D structure, and the structural heterogeneity exhibited by some PBVs (108–111). The last consideration, epitope size, consists of constraints that are somewhat vague. Conceivably, any molecule or portion of a larger antigen that is soluble and large enough to initiate a BCR signaling cascade can be considered a BCR epitope. As such, there is no upper limit on antigen size outside of reasonable physiological constraints and any lower size limitation most likely coincides with major histocompatibility complex (MHC) binding limitations (where 10–20 mer polypeptides are necessary). BCR epitope size, on the other hand, is only limited by the diameter of recognition on a BCR paratope (~40 Å). A lower threshold for epitope size must also exist, but where this falls is subjectively based on the extent of non-covalent interaction between paratope and epitope.

### Chemical Conjugation of BCR Epitope to PBV

Inherently non-immunogenic molecules that fall below the antigen size threshold but have the capacity to sufficiently interact with antibody paratope can be made immunogenic via covalent attachment to a larger immunogen. These molecules, dubbed haptens, are the premise behind conjugate PBVs. It is conceivable that any molecule could be forced into the role of a BCR epitope using this approach. This rationale is exemplified by conjugate PBVs that have successfully elicited humoral immune responses against glycans, self-antigens, and drugs of abuse (112–114). When designing a conjugate PBV, the most important considerations are choice of protein carrier and choice of hapten. Choice of protein carrier is crucial because it determines the number of potential conjugation sites and where they are located. These properties affect (1) conjugation efficiency, (2) conjugation number, and (3) masking of carrier protein AIRs. This, in turn, has a profound influence on the ability of conjugate PBVs to redirect the humoral immune response away from carrier protein and toward hapten (115). Additionally, the number of MHC class II epitopes found within carrier protein primary sequence and their affinity for receptors can potentiate humoral immune response through (1) the targeting of specific human leukocyte antigen (HLA) haplotypes within a population and (2) the activation of helper T (T_H_) cells (30, 116). This applies more to the targeting of TCR, however, and as such will be discussed in future sections. Choice of hapten has been shown to have a sizeable impact on the number and binding characteristics of antibodies elicited by conjugate PBVs (117, 118). This effect is exemplified in nicotine vaccines by the difference in efficacy observed when only the attachment position to nicotine is changed (119). Consequently, hapten design has been extensively investigated for PBV formulations targeting drugs of abuse, as these vaccines require large quantities of high affinity antibodies in order to effectively sequester drug in the blood and extracellular fluid (120, 121).

### Genetic Fusion of BCR Epitope to PBV

A similar embodiment to the conjugate PBV is the chimeric, fusion PBV. By definition, the flagellin and lipoprotein fusion proteins described previously can be considered chimeric PBVs. However, within this context, the term most often refers to immunogens that have been rationally modified with recombinant epitopes. With this approach, instead of chemically attaching epitopes to immunogen surface, genes are recombined such that conformational or linear polypeptide epitopes are inserted within PBV immunodominant regions. These regions should be surface exposed if the intent is to activate a humoral immune response (i.e., AIRs). If epitope insertions can be made within AIRs without negatively influencing protein folding, it ultimately results in B cell responses being redirected away from the previous AIR and toward the introduced epitope. This concept was demonstrated by Gillam et al. when they reported increased antibody titers to recombinantly inserted YSNIGVCK epitope and decreased titers to hepatitis B core antigen (HBcAg) VLP when evaluating a porcine epidemic diarrhea PBV in mice (122). A maximal insert size exists for all AIRs in which proper protein folding can still be accommodated. Successful inserts within HBcAg have been reported at >200 residues, whereas inserts within HPV 16 L1 protein, even when at the C-terminus, rarely exceed 60 residues without negatively influencing PBV structure (123, 124). Additionally, Varsani et al. observed that epitopes of identical length had variable effects on the ability of chimeric HPV 16 L1 PBVs to form VLPs when they were inserted within different AIRs (125). Together, these results indicate that maximum insert size is variable and largely influenced by the properties of the protein, the insert, and the insert location. It also appears that larger epitopes may influence protein structure less than smaller ones in some cases, but this is not the norm (126).

The fusion approach to targeting epitopes is most commonly employed using VLPs as scaffolding, a technology which comprises the assemblage of multiple protein copies all containing the same immunodominant regions. In this way, high epitope densities per antigen can be achieved without sacrificing the intrinsic, immunogenic advantages provided by VLP shape, size, and structure (4). Chimeric VLP PBVs have been successfully developed to target a variety of infections that plague humans and livestock species. Examples include a HPV 16 L1-based vaccine targeting influenza A virus (126), a MS2-based vaccine targeting HIV (127), and a HBcAg-based vaccine targeting porcine reproductive and respiratory syndrome virus (128), though many other studies implementing this technology exist (129). Chimeric, fusion PBVs have also been specially designed to convey cross-protective immunity via the insertion of broadly neutralizing and/or multiple BCR epitopes (130). It is important to note, however, that VLP-forming proteins are not the only proteins that can support this approach. Alternatively, toxoid proteins have also been used in chimeric vaccines targeting insert-specific antibody production, though much less frequently than their VLP counterparts as they do not offer many of the same advantages mentioned earlier (131–134).

### Carrier Induced Epitopic Suppression

Recent strides in vaccine development have been accompanied by increased interest in conjugate and chimeric BCR epitope PBVs. When combined, they present an outstanding opportunity for vaccinologists to generate vaccines that can target nearly all conceivable chemicals, biologicals, and polypeptides. In addition, they provide a means through which epitope densities can be increased on antigen surface such that BCR cross-linking and subsequent T cell-independent activation of B cells becomes more likely. However, the promise of a “vaccine for anything” provided by conjugate and chimeric PBVs has elicited various complications. Of these complications, carrier-induced epitopic suppression (CIES) is the most noteworthy issue. Both conjugate and chimeric PBVs require the use of existing immunogens as carrier proteins in order to elicit an immune response. This, along with the fact that there are a limited number of suitable immunogens available for use in vaccine formulations due to factors such as immunogenicity, toxicity, stability, and accessibility, leads to a situation where a select group of “preferred” immunogens are most often used. CIES refers to a phenomenon that occurs when the same immunogen is used in sequential, independent vaccine administrations targeting different epitopes, resulting in the sequestration, elimination, and/or inhibition of vaccine response to target epitope by pre-existing, immunogen specific antibodies and lymphocytes (135). As expected, this can lead to inhibition of hapten-specific lymphocyte recognition of vaccine and an ultimate reduction in vaccine efficacy (136).

Though CIES is likely to always occur to some extent when simply boosting a conjugate or chimeric PBV, prevention of antibody specificity for new vaccines is paramount to the creation of more successful immunizations (137). It has been shown that CIES can be overcome by increasing vaccine dosage and/or including more booster injections within a vaccine regimen. More interesting, however, is the positive effect that increasing hapten density has had on the occurrence of CIES, presumably through the crowding of carrier-specific BCR epitopes (138). Interruption, removal, or blocking of AIRs in chimeric VLP formulations via recombinant and/or chemical means has been shown to improve immunogenicity toward target epitopes and minimize immunogenicity toward carrier protein, further corroborating this presumption (122, 139, 140). However, chemical modification, such as PEGylation, is largely non-specific, thus resulting in unpredictable outcomes for vaccines. For this reason, it is mainly employed to reduce therapeutic protein antigenicity (141–144). Ideally, immunogen surface should be considered for each individual vaccine in order to simultaneously direct antibody-mediated immune response toward important epitopes and prevent pre-existing antibody recognition of carrier-specific immunodominant regions.

## Targeting T Cell Receptors

### PBVs and the T Cell

The most critical component in any adaptive immune response is arguably the T cell, as it serves as a key facilitator of both cell-mediated and humoral immunity. T cells assist with B cell maturation (T_H_ cells), destroy infected and malfunctioning cells (cytotoxic T (T_C_) cells), prevent T cell autoreactivity and terminate T cell activity at the end of an immune response (regulatory T (T_Reg_) cells), provide tissue, effector, central, and virtual antigen memory (memory T (T_M_) cells), in addition to assuming many other roles (natural killer T (NKT) cells, mucosal associated invariant T (MAIT) cells, and gamma delta T (T_γσ_) cells) (145). At the heart of this broad functionality lies the TCR, the associated MHCs (class I and class II), and TCR epitopes. When a PBV is administered, cellular processing of antigen leads to MHC display of small, linear, antigen-derived peptides, also known as TCR epitopes, on cell surface. TCR CDR recognition of these MHC-peptide complexes, in turn, leads to T cell activation and proliferation. More specifically, activation of CD8^+^ T_C_ cells is facilitated by MHC class I molecules whereas activation of CD4^+^ T_H_ and T_Reg_ cells is facilitated by MHC class II molecules. CD4^+^ and CD8^+^ T_M_ cells can be activated via either MHC class I or MHC class II pathway (28, 146). Many antigen-associated factors influence the type and magnitude of T cell response. The most important of these factors include antigen uptake, localization, processing, and T cell epitope content.

### The Impact of APC Uptake of PBV on T Cell Activation

The first step in the T cell activation process is uptake of antigen by cells that express MHC molecules. For the purposes of PBVs, this process is generally orchestrated by specialized APCs, though many other cell types can participate in the event of cellular damage or infection. In fact, the MHC class I pathway can be initiated by any nucleated cell. Alternately, activation of the MHC class II pathway can only be achieved after phagocytosis of antigen by APCs (147). For this reason, fusion of PBVs to antibodies and antibody fragments specific for APC surface markers has been extensively investigated as means of potentiating both helper and cytotoxic T cell activation. This approach has routinely demonstrated success, with antibody-mediated, APC targeting generally resulting in a subsequent increase in cellular and humoral mediated immune response (148–156). PBVs have also aimed to rationally target T cell activation through the selective incorporation of ligands specific for specialized receptors found on APCs. These receptors include PRRs such as integrins and CLRs, MHC class II molecules, and Fcγ receptors, among others. However, most research has focused on CLRs such as DC-SIGN, langerin, and the DECTIN-1 subfamily (157, 158). This approach presents a major advantage over the old method of tethered antibody mediated targeting in that incorporation of glycans specific for CLRs do not require any additional steps when appropriate expression hosts are used.

### The Impact of Cellular Localization of PBV Within APCs on T Cell Activation

Antigen localization is one of the major controlling factors behind the type of T cell response initiated by exogenously administered PBVs. MHC class I pathway requires internalization and processing of antigen into short peptide sequences (8–10 amino acids) within the cytoplasm. In contrast, MHC class II pathway requires phagocytosis of antigen by APCs and subsequent lysosomal processing into somewhat longer peptide sequences (15–24 amino acids) (147). As such, attempts have been made in the past to direct PBV to cytoplasm (to target MHC class I pathway) or lysosome (to target MHC class II pathway) using co-administration and fusion protein approaches. Results have been mixed, with some studies observing increases in pathway-specific immune response and others showing no improvement. Targeting lysosomal degradation by tethering antigen to lysosome-associated membrane protein (LAMP) generally has shown no significant increase in antigen-specific antibody population and only marginal increases in CD4+ T cell population (159, 160). Invariant chain (Ii) has also been fused to antigen in an attempt to target lysosome and subsequent MHC class II pathway. While fusion of full-length Ii to endogenous antigen results in inconsistent activation of CD4+ T cells, most likely due to the presence of class II associated Ii peptide (CLIP) on the C-terminus, fusion of Ii derivatives accounting for various lengths of N-terminal sequence have been shown to effectively direct antigen to lysosome (161, 162). Exogenously administered fusion proteins that have been tagged with an Ii portion thought to assist with MHC class II loading (LRMK, Ii-Key) have also been explored, resulting in potentiation of both CD4+ and CD8+ T cell immune response (163–168). Finally, since APCs are the only cell type capable of processing antigen via the MHC class II pathway, the targeting of APC uptake is also a viable means of targeting CD4+ T cell activation. This approach to controlling antigen localization has generally proven to be non-specific to humoral or cytotoxic pathway, however, as it dually potentiates activation of both CD4+ and CD8+ T cells (148–156).

### The Impact of PBV Stability on APC Processing and T Cell Activation

APCs, specifically dendritic cells (DCs), are unique in their ability to activate T_C_ cells after exposure to extracellular antigen. This cross-presentation (CP) of antigen is key to the initiation of cellular immunity to many cancers, viruses, and exogenously administered PBVs (169). The majority of CP in APCs is due to antigen stability and slowed lysosomal digestion, though other factors, such as the vacuolar pathway, cell maturation stage, and immunostimulatory environment, do play a role (170–177). PBVs can be engineered to improve stability, as has been demonstrated by the introduction of inter-capsomeric di-sulfide bonds within HBcAg VLPs and MS2 bacteriophage VLPs (122, 178–180). The effect that this engineered stability has on MHC pathway, however, has not been thoroughly investigated, though it appears that stabilization of antigen promotes CP and the activation of T_C_ cells as destabilization has been shown to reduce cross-presentation efficiency (181). One study conducted by Schliehe et al. was able to observe that recombinant fusion of ubiquitin (Ub) or Ub-like modifier Fat 10 to vaccinia virus nucleoprotein (NP) resulted in abrogated protein stability and T_C_ and T_M_ cell response to select immunodominant NP epitopes (174). Another study conducted by Delamarre et al. showed that the slight structural difference between RNase-A and RNase-S (where RNase-S has a peptide bond cleaved between A 20 and S 21) caused RNase-S to be more susceptible to lysosomal proteolysis *in vitro* and that this susceptibility ultimately resulted in reduced ability for mice to mount a humoral immune response upon vaccination. The T cell activation upon incubation of RNase-A or RNase-S with splenocytes harvested from RNase-S vaccinated mice was near zero, however, which seems to indicate that RNase-S failed to reach APCs upon *in vivo* administration (182). When considering MHC class II pathway, increased antigen complexity has been shown to slow *in vitro* DC processing and decrease *in vivo* antibody response (183). For example, So et al. observed that the stability of two recombinantly modified hen-egg lysozyme proteins (one with a deleted di-sulfide bond and the other with a selectively added intramolecular ester bond) was inversely correlated with CD4+ T cell activation and both cytokine and antibody production in a mouse model (184). When paired with evidence suggesting that these observations are not due to differences in T cell epitope content, these results indicate that PBV stability plays a crucial role in managing the adaptive immune system during an immune response (173, 185). Whether or not the extent of PBV processing is orchestrated by the cell with the intent of modulating humoral and cellular immune responses, however, remains to be seen.

### The Importance of the T Cell Epitopes in PBV Efficacy and Design

After cellular processing of antigen, presentation of MHC-peptide complex to TCR can't take place if there are no antigen-derived, T cell epitopes specific for the MHC molecules expressed by the cell, as only MHC-peptide complexes can bind TCRs and activate T cells (28). For this reason, toxoid proteins and toxoid protein derivatives have been used for many decades in the form of adjuvants and fusion proteins to supplement antigens that are lacking in T cell response. Eventually, the improved immunogenicity of these combinations was associated with the density and promiscuity of T cell epitopes contained within the toxoid proteins (186). Since this realization, scientists have searched for “universal” T cell epitopes (UTEs), or rather epitopes that can bind with a large portion of the MHC phenotypes found within the human population (187). This search has been rewarding, with UTEs having been discovered in a plethora of pathogen-associated proteins.

UTEs come in two forms; those that activate T_H_ cells via MHC class II pathway (UhTEs) and those that active T_C_ cells via MHC class I pathway (UcTEs). Synthetic UhTEs, such as the Pan DR Epitope (PADRE) peptides, have also been engineered. Their development has typically been based on the fact that properties of residues at certain positions within an epitope are more important for MHC binding than others (188, 189). This principle has also led to the construction of data-trained algorithms that can help predict MHC binding preferences (190, 191). As such, it is now possible to dissect an antigen *in silico*, identifying key sequences that are most likely to serve as epitopes for MHC molecules. Taking this approach to PBV design now makes it possible to direct the immune system toward either a cell-mediated or humoral response and increase *in vivo* T cell response to PBV by targeting more MHC phenotypes. Ultimately, the use of these algorithms is paving the way toward vaccine designs that target predominant HLA haplotypes, further broadening the range of subjects that respond to PBVs (192–195).

Though TCRs aren't subjected to the same post-activation, somatic hypermutation that BCRs are, they do undergo VDJ recombination and as such their affinity for MHC-peptide complex should theoretically be random (28). BCR specificity is not completely random, however, and considering the similarity between BCR and TCR CDRs, it is reasonable to assume that TCRs, like BCRs, may also preferentially bind polypeptide motifs displaying certain quantifiable properties. This hypothesis was recently supported by Chowell et al. when they observed that TCR contact residues exhibit a strong bias for hydrophobic amino acids contained within MHC class I epitopes (196). They postulated that this bias was due to the favorable thermodynamics associated with TCR covering hydrophobic residues on MHC-epitope complexes. Parrish et al. also presented evidence that TCRs are germline encoded to have intrinsic specificity for unloaded MHC molecules (largely independent of allele, class, and polypeptide sequence) (197). Furthermore, the existence of superantigens (SAgs), atypical immunogens that allosterically interact with MHC class II molecules, crosslink the variable region on TCR β-chain, and have the capacity to activate up to 20,000-fold more T cells than are activated by typical antigens, provides additional evidence for this principle. The binding of SAgs by TCR is largely non-specific when compared with interactions between TCR and peptide-loaded MHC molecules, indicating that, at least under certain circumstances, structurally distinct TCRs have a propensity to bind specific antigenic motifs (198). Together, these results strongly suggest that interactions between TCR and MHC-epitope complexes are not random and that there may be opportunities in the future to develop algorithms, like those modeling MHC-epitope interactions, that can predict TCR binding of MHC-epitope complexes.

### Targeting TCR via Recombinant and Conjugate Approaches

Many groups have targeted T cell activation via chimeric or covalent attachment of naturally, synthetically, or computationally derived UTEs to protein and peptide-based vaccines. A recombinantly modified rabbit haemorrhagic disease virus-like particle-based vaccine developed by Jemon et al. that incorporated the universal T cell epitope PADRE and an MHC I-restricted epitope derived from the HPV 16 E6 protein (aa 48–57) showed promise as an anaphylactic HPV 16 vaccine when it reduced the tumor burden and improved the survival time of HPV tumor-bearing mice (199). In another study, Percival-Alwyn et al. observed that CD1 mice were able to mount an autoimmune response to self-protein ST2 only once it had been tethered to the A fragment of DT (DTA) or dual T_H_ epitopes derived from TT. Interestingly, the dual epitope performed better than DTA at eliciting autoimmunity when comparing antibody titer differentials between ST2 and control fusion proteins. When considering the size of the two inserts, this result supports the earlier consideration that inserts have the capacity to redirect immune response away from target epitopes (in this case, antibody responses seem to have been redirected away from ST2 and toward DTA) (200). The epitome of the epitopic approach to vaccination was explored by Wu et al. when their group fused a single BCR epitope derived from epidermal growth factor receptor (EGFR, aa 237–267) to a UTE derived from measles virus fusion protein (MVF, aa 288–302) and saw a sizeable antibody response in a mouse model (201). A computational approach to vaccine design was evaluated by Hurtgen et al. when their group used software to predict the UTEs found within three *Coccidioides posadasii* antigens, Pep1, Amn1, and PIb. Using *in vitro* assays to screen for immunogenicity and MHC II affinity, five epitopes were selected for incorporation within a recombinant epitope-based vaccine (along with murine Ii-Key and spacer sequences) and subsequently evaluated in an HLA-DR4 transgenic mouse model. Recall epitope assay indicated that 4 of the 5 epitopes successfully activated T cells and challenge assay results showed early activation of T_H_ cells, elevated interferon and interleukin expression, and prolonged survival rates in vaccinated mice (202). Other studies have observed similar, positive results when employing this approach to targeting T cell activation (203–206).

### Limitations of Experimentally and Computationally Determined UTEs

UTEs provide a rational means of potentiating humoral and/or cytotoxic immunogenicity of both peptide and protein-based vaccines. Their incorporation into PBV designs could also provide a means of overcoming the effects T cell competition when common antigens are used as vaccine (207). Experimental discovery of UTEs, however, is made difficult by the fact that the HLA system is one of the most variable gene complexes found in humans. For example, at least 3.82e8 combinations exist for MHC class I-associated alleles, and that number is far greater for MHC class II-associated alleles (208, 209). Additionally, the HLA complex shows considerable difference between humans and common animal models such as mice (210). As such, computational approaches to predicting UTEs for specific MHC phenotypes are oftentimes plagued by insufficient amounts of raw data on MHC-epitope interactions, data that are needed in order to establish and validate prediction algorithms. As a result, most methods' predictions can establish what peptide stretches qualify as binders and non-binders, but for the most part these predictions fall short of the quality needed to become the basis for sizeable PBV decisions (185). Ultimately, however, predictions will improve as more data become available for algorithm training and calibration, making it likely that epitope prediction software will become a common tool in the vaccinology toolbox at some point in the future.

### PBVs, T Cells, and Their Implications in Cancer Therapy

PBVs targeting cancer are particularly important when considering antigen processing and MHC epitope presentation. This is because, due to the intracellular nature of most TAAs, the activation of both CD4+ and CD8+ T cells are critical to their success. This makes targeting optimal antigen processing a difficult endeavor, as a balance between MHC class I and MHC class II epitope content must be considered. Additionally, since TAAs are generally endogenous, it is typical for the immune system to have already established a degree of central and peripheral tolerance toward them, making epitope-specific immunostimulation more difficult (211). Multi-epitope vaccines containing both MHC class I and MHC class II epitopes that have been defined using observation and/or predictions have been a promising development in the field of cancer immunotherapeutic. Xiang et al. explored the idea when their group strung together groups of UcTEs and UhTEs and chemically conjugated them to the surface of a polystyrene nanovaccine platform in their search for polypeptide-based, therapeutic HPV, survivin, and Wilms Tumor antigen 1 (WT1) vaccines (212). Results from this study indicated that incorporation of UhTEs with cancer-associated UcTEs can assist with the UcTEs' immunogenicity, though it is interesting that this result was not observed upon the incorporation of CpG adjuvant and that simpler epitope-based vaccines were more effective under certain conditions. In another example of this approach, Lin et al. evaluated the efficacy of a multi-epitope, cancer PBV consisting of predicted T_H_, T_C_, and B cell epitopes of the LMP2 protein (a TAA found in EBV-associated cancers) (213). Though therapeutic efficacy of this vaccine was not evaluated, inoculation of BALB/c mice resulted in sizeable PBV-specific IgG, IgA, and CTL response. Additional examples also exist, and it is likely that many more examples will arise in the years to come (214).

## Additional Considerations

### PBV Safety Considerations

One of the primary reasons PBVs were explored as an alternative to live attenuated and inactivated vaccines in the past was their improved safety profile. This, however, was before genetic modification of antigen had become feasible. Today, modifications made to antigen structure can influence PBV safety profile both positively and negatively. For example, attempts to improve PBV immunogenicity via the many fusion protein approaches previously described could backfire in the form of adverse events, such as cytokine storm and molecular mimicry (autoimmunity instigated by epitopic similarities between foreign and self-immunogens) (74, 215). On the opposite end of the spectrum, the need for safer carrier proteins in subunit vaccine formulations has led to modifications being made to many bacterial toxins with hopes of finding safer alternatives (19, 22). Ultimately, the extreme variations observed in PBV efficacy and safety when even the smallest changes are made in antigen structure leads to a situation where safety evaluation becomes all-the-more critical throughout the vaccine development process. This is compounded by the fact that vaccines are generally administered to healthy subjects, making adverse events even less acceptable when compared with other medicines being developed to maintain non-maleficence. As such, additional steps should be made beginning with the very first animal studies when assessing the safety and efficacy profiles of modified PBVs outside of the standard establishment of correlates of protection and the full characterization of vaccine formulation.

### Animal Model Considerations

PBVs must first prove themselves in numerous animal studies before they can be used in humans. Unfortunately, however, immunological and physiological interspecies differences make it unwise to extrapolate pre-clinical results to human studies even when using the most optimal animal model for the PBV being developed (216–219). Outside of obvious issues with dose scaling, a perfect example of this is illustrated when one considers that the animal models typically used in vaccine research are inbred to the point of isogenecity. This can be considered determinantal when performing vaccine research because some of the most diverse genes in humans, such as those located on the HLA gene complex, code for important immune molecules that have a major impact on the nature and magnitude of immune responses to antigen and vaccine (220). Even when using humanized HLA transgenic (Tg) mice as an animal model in vaccine studies, cytotoxic T cell epitope recognition concordance rates with humans have only been reported at 47% for vaccinia virus (following immunization with full virus) and 68% for HIV (following immunization with peptide) (221, 222). Though the sample sizes for these studies were small, these results demonstrate that both epitope recognition and antigen processing are quite different between animal models and humans. The effectiveness of some adjuvants can also vary widely between species, making it difficult to ascertain the true value of vaccine efficacy studies done in animals. For example, the antibody response to HBcAg adjuvanted with oil-in-water MF59 adjuvant system is nearly 10 times more potent in humans than in mice and approximately 4 times more potent in humans than in baboons (223). Summarily, the correlative utility of potential animal models when assessing PBV efficacy, especially when investigating the effects of TCR epitopes and adjuvants, should be carefully considered before *in vivo* testing in order to insure the best translation from animal to human success. Only in this way can we hope to consistently elucidate the impact of PBV modification and formulation on vaccine safety and performance in humans. It is safe to assume, however, that there will always been some level of uncertainty prior to starting *in vivo* studies.

### Additional Stability Considerations

**Figure 4:**
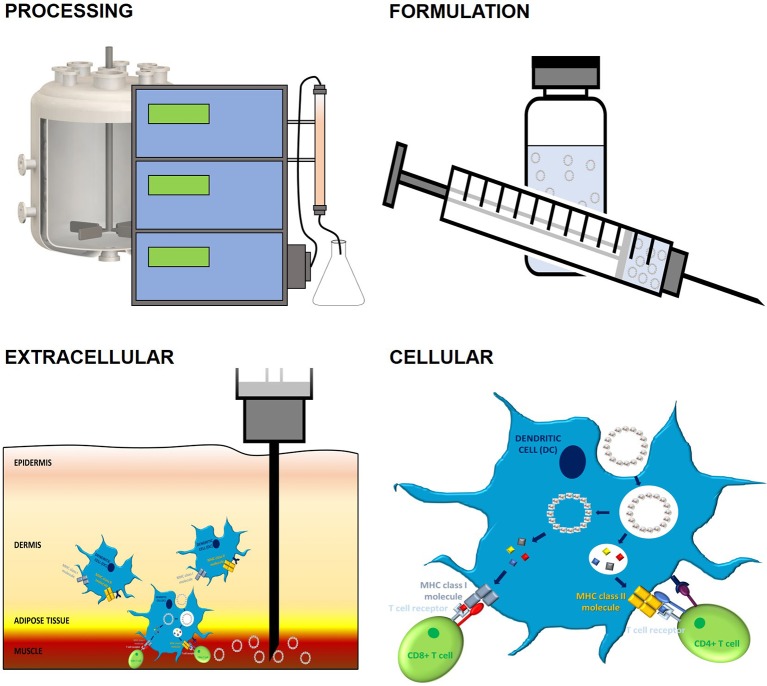
The impact of PBV stability on immune response. PBV stability has a profound impact on conformation, immunogenicity, and vaccination outcome. Outside of inherent fold stability that's dictated by protein primary structure, many factors contribute to final PBV conformation and stability. Upstream and downstream processes, such as expression and purification, have a sizeable impact on the capacity of PBV to form higher-order structures and can ultimately lead to unintended PBV surface modification, aggregation, and/or decomposition. These issues can also present during formulation as a result of protein-protein, protein-adjuvant, and protein-container interactions. Incompatibilities between physiological conditions and PBV formulation can result in poor extracellular stability, a phenomenon that often presents as excessive local inflammation and poor transport of antigen to secondary lymphoid organs. Finally, cellular stability, which is a function of all the factors mentioned previously, largely dictates MHC processing and the nature of the immune response orchestrated by APCs (cellular vs. humoral, Th1 vs. Th2 vs. Th17, etc.). The 3D protein structure for HBcAg used in this image was rendered in PyMOL 2.3.0 and accessed via the Protein Data Bank (14, 18, 29).

As has already been mentioned, conformation and stability play an important role in the immunogenicity of PBVs. This importance, however, goes beyond the ability of APCs to cross-present antigen (Figure 4). Recombinant proteins, especially when expressed in lower-order systems and/or as inclusion bodies, are notorious for their inclination to misfold (224). This often results in unstable, heterogenous mixtures of protein-derived particles that may (1) inefficiently present BCR epitopes, (2) denature prior to encountering immune cells, or (3) fail to degrade within endosomal compartments (173). Many components commonly found in PBV formulations can also negatively affect conformation and stability. Specifically, alum, oil-in-water, and TLR agonist adjuvants have the potential to cause PBV structural changes due to electrostatic, hydrophobic, and/or coordination interactions with amino acid side chains (225). Since these immunostimulatory molecules are regularly necessary to improve PBV efficacy and help prevent peripheral tolerance, an event that occurs when the adaptive immune system encounters antigen in low doses or outside of an inflammatory setting, simple removal is not an option. Protein-protein interactions can also become a problem when PBVs are rich in reactive side chains. Stabilizing agents such as polysorbate 80 and sucrose can be employed to help prevent protein-protein and protein-adjuvant interactions in PBV formulations, but these molecules also have the capacity to detrimentally interact with certain amino acid side chains via glycation and oxidation (226). Protein aggregation, however, does not always have a negative impact on immunogenicity. Aggregation of therapeutic proteins has been shown to augment the formation of anti-drug antibodies in a phenomenon that is likely caused when immune epitopes/ligands become easier to access/process (227). Additionally, it seems that conjugate PBVs may benefit from some level of aggregation. This is because, while the conjugation approach to PBV design insures proper BCR epitope presentation, aggregation has also been shown to improve DC uptake by more than 3 × and facilitate DC drainage to lymph nodes (54). Of course, improving PBV immunogenicity via engineered aggregation would not be beneficial when employing a chimeric approach to PBV design as the aggregated vaccine structure would most likely present immunological determinants that are much different from those that were intended. Evidently, many important structural and formulative considerations must be made when designing modified PBVs.

### PBV Targeting Strategies for Antigenically Variable Pathogens and Cancers

The development of PBVs targeting antigenically variable pathogens (AVPs) and immune-resistant cancers (ARCs) is complicated by the effects of epitope masking, antigenic drift (the natural accumulation of mutations within existing immunological determinants), antigenic shift (the natural recombination of immunological determinants between independent influenza A strains), and immunoediting (when the natural anti-tumor activity of the immune system puts pressure on oncogenic cells to resist/prevent immunorecognition). These events are characterized by mutations within antigenic determinants that make it difficult for an adaptive immune response to hone in on antigen (228). Multiple strategies to overcoming these complications have been investigated.

For PBVs targeting influenza, the most common approach is biannual reformulation of multivalent, recombinant hemagglutinin PBV such that the flu strains predicted to have the most impact on public health are targeted (229). Unfortunately, this approach to combating AVPs cannot be applied to HIV due to the severity of complications associated with and the chronic nature of HIV infection. One exceptionally promising vaccination approach to protecting against HIV, however, appears to be the reverse engineering of PBVs based on broadly neutralizing antibody (bNAb) populations either found in seropositive individuals or discovered by using massively parallel mapping techniques (230, 231). The premise behind this approach is simple. During the initial stages of HIV infection, the war between mutating antigen and adaptive immune response results in the generation of bNAbs that have been shown to confer powerful protection upon passive immunization in various animal models (232). Intuitively, it should be possible to use structural vaccinology principles and reverse engineer PBVs that can elicit protective antibodies with similar specificities to previously identified bNAbs using an active immunization approach. Scientists hoping to use this approach to develop an effective, prophylactic HIV PBV have identified five key sites associated with bNAbs and work is underway to construct PBVs that can target these vulnerable sites (233–235).

It is worth noting that any active vaccination attempt against HIV will most likely have to consider more than just BCR epitope presentation in order to be effective. This is because, in addition to masking and mutating BCR epitopes, HIV is notorious for its ability to escape immune recognition via alterations in TCR epitopes, specifically those that are recognized by MHC class I molecules (236). A *post-hoc* analysis of the most successful HIV vaccine study conducted to date, which used an RV144 pox virus prime, recombinant HIV-1 gp120 (rgp120) boosts, and had an estimated 31% overall protection rate, supports this conclusion. Within the vaccinated population, the HLA A^*^02 genotype was a marker for success, with A^*^02^+^ individuals showing significantly greater protection than A^*^02^−^ individuals (54 vs. 3% effective). Other studies have made similar observations (237, 238). These results not only illuminate the importance of making HLA-targeting considerations in the PBV design process, but also indicate how impactful MHC escape mutations can be when using PBVs to protect against AVPs (even when only one set of HLA-specific epitopes is affected). In this same study there was also a significant increase in vaccine efficacy within the A^*^02^+^ population when the presence of a lysine at position 169 in the V2 region of rgp120 was factored in (74 vs. 15% effective) (239). Since this region of the HIV-1 proteome is known as one of the five key bNAb eliciting sites, this result further highlights the challenge associated with targeting AVPs via antibody-mediated approaches (240). Ultimately, it is very possible that the success of future HIV vaccines will be dictated by how well-antigenic determinants can be targeted and controlled using PBV formulations.

Much like viruses, cancers are subjected to selective pressures by the immune system. The majority of malignant cells are recognized and eliminated during immunosurveillance via a variety of effector mechanisms. However, these cells are continuously incentivized to alter and/or hide their antigenic determinants in order to survive and proliferate. As a result, malignant cells that survive immunosurveillance and establish tumors frequently express specific, targetable TAAs that have evolved to maximize survival and minimize immunorecognition (241). This makes the targeting of TAAs via PBVs a tangible option when conceptualizing cancer immunotherapeutics, especially when used in concert with therapies that abrogate the immunosuppressive nature of the tumor microenvironment (e.g., the immune checkpoint inhibitors (ICIs) anti-PD-1 and anti-CTLA-4 and the co-stimulatory molecules (CSMs) anti-CD137 and anti-OX40) (242, 243). Unfortunately, the combination of epitope and multi-epitope vaccines with ICIs has thus far produced largely unimpressive results (244). Additionally, little information could be found on the marriage of full-length PBVs and these immunomodulators (most cancer vaccines utilizing this approach are cellular, DNA, or viral vector-based). The lack of interest in applying this approach to PBVs could be due to the nature of the immune system (cellular, DNA, and viral vector-based vaccines are generally better than PBVs at eliciting CD8+ responses) and/or the failure of past PBV cancer vaccines to advance through clinical trials (245).

### The Chimeric-Conjugate Approach

Residues commonly used for chemical crosslinking can be recombinantly incorporated onto protein surface in order to increase conjugation capacity of PBV and ultimately improve vaccine performance (chimeric-conjugate approach). In one study evaluating this approach, researchers were able to recombinantly substitute threonine-15 of the MS2 coat protein with a cysteine residue without influencing folding efficiency (246). Another study observed that replacement of proline-79 and alanine-80 with the peptide GGKGG within the immunodominant region of modified HBcAg resulted in increased titers against conjugated M2 and improved survival rates upon viral challenge when compared to WT HBcAg that had been recombinantly fused to the same protein (247). Similar research using lysine-modified tobacco mosaic virus (TMV) coat protein virus-like particles (VLPs) reported an increase in immunogenicity when compared to co-administered antigen and VLP (248) and another study that used lysine-modified and cysteine-modified TMV VLPs observed that coat protein assembly around RNA scaffold could be modulated by altering the ratio of the two mutant proteins during *in vitro* assembly (249). Together, these results indicate that a chimeric-conjugate approach could easily be employed to incorporate epitope and/or PAMP ligands with PBV, though more research will need to be done if we wish to fully understand the effect of this method on loading capacity and immunogenicity.

### Additional PBV Modification Techniques

Additional PBV modification techniques exist that could be employed to modulate immune responses and target the key immune receptors discussed here. The incorporation of unnatural amino acids (UAAs), such as p-nitrophenylalanine, within PBV structure has been used to overcome autoimmune tolerance in vaccines against RANKL and TNFα (250, 251). Incorporation of the UAA azidohomoalanine have also been used to enable click chemistry on PBV surface (252). As opposed to traditional carbodiimide and maleimide conjugation techniques, click chemistry allows for rapid reaction kinetics, selective ligand attachment, and high yields of successfully utilized attachment sites (253). Sortase-mediated conjugation allows for the highly specific attachment of LPETG(G) tagged molecules to PBV bearing recombinantly inserted multi-glycine stretches, though coupling efficiencies (~30%) tend to be low when using this technique (254, 255). Polyhistidine (pHis) tags are well-known for ability to interact with immobilized metal ions in affinity chromatography applications. At least one research group has attempted to utilize this interaction when conjugating nickel-loaded, tris-nitrilotriacetic acid (tNTA) ligand to norovirus (NoV) VLPs that had been C-terminally modified with pHis tags of various lengths. Results confirmed the utility of the approach as a means of attaching ligand to PBV surface and suggested that the degree of tNTA loading correlated with the number of NoV VLP subunits that contained a pHis tag (256). It may be unwise to employ this conjugation technique when designing future vaccines, however, as nickel has been established as both an allergen and a carcinogen (257). Protein affinity-tag interactions have also been explored as an alternative means of tethering ligand to PBV (258). In one example of this approach, Thrane et al. screened multiple chimeric HPV 16 L1 vaccines that recombinantly displayed a biotin acceptor sequence on one of the protein's surface loops. Insertion did not prevent formation of VLPs in all but one construct, and biotinylation and subsequent attachment of cVLP (HI loop insert) with monovalent streptavidin (mSA)-fused VAR2CSA ligand (a portion of *Plasmodium falciparum* erythrocyte membrane protein 1 that can be implicated in malaria pathogenicity) showed significant improvement in antigenicity over free ligand early on in vaccination timeline and similar efficacy in final blood draws when the two PBVs were evaluated in a mouse model (259). Clearly, the vaccinologist toolbox is deep when it comes to ways of tethering ligand to PBV.

## Conclusions

Modified PBVs present an effective means of overcoming many of the limitations encountered by today's vaccines. First, the addition of PAMP ligands to PBV structure insures that sufficient immune activation signals are co-delivered with vaccine upon administration. PBV modifications of this type have been shown to increase overall vaccine immunogenicity and should reduce the likelihood of initiating immune tolerance. Second, recombinant and/or chemical modification of PBVs with BCR epitopes can modulate humoral immune response to vaccine. Specifically, this type of modification makes it possible to (1) direct antibody specificity toward select epitopes, (2) design cross-reactive vaccines that can neutralize multiple epitopes, and (3) prevent CIES when the subject has previously been exposed to antigen. Third, the recombinant incorporation of TCR epitopes within PBV structure is useful in that it can (1) allow targeting of specific HLA genotypes/MHC phenotypes, (2) influence the type of cellular immune response that's initiated, and (3) overcome the effects of T cell antigenic competition. Finally, modifications can be made to PBV primary structure that influence stability and cellular uptake, both important factors when it comes to the magnitude and type of immune response initiated.

Limitations associated with PBVs also exist. These are generally structural in nature and can result in the negative modulation of both safety and efficacy. First, it is possible for modification and/or misfolding of PBV to result in the incorporation of unintended PAMPs and antigenemic determinants, an effect that could lead to issues with immunotoxicity and/or autoimmunity. Second, the extensive structural variability cognate with PBV production processes and the general weakness of animal models as success correlates also makes it difficult and expensive to quantify just how well PBVs will work prior to direct evaluation in their target species. Along these same lines, issues with protein stability and interactions between vaccine species when making modifications to PBV formulations can add an additional confounding element to the design process, as the margins between protective immunity and immune tolerance are often very fine. Lastly, AVPs present an additional challenge when concerning the utility of PBVs due to the difficulty of targeting ever-changing, antigenic determinants.

From prophylactic use aimed at preventing hard-to-treat diseases such as HIV to anaphylactic use aimed at alleviating chronic conditions such as addiction, allergies, and autoimmunity, the utility of PBVs has advanced well-beyond what was previously imagined possible. Here, we have described the mechanisms behind PBV immunogenicity and listed many structural modifications that have been explored in the past as a means of modulating and/or potentiating PBV *in vivo* effects. The results from these studies have shown promise on a case-by-case basis, but thus far we have yet to realize the dream of a silver bullet, modified, PBV vaccination approach that would allow the rational targeting of any epitope in any species with a high response rate and without complications. It is possible that we never will, due to the complexity of the immune system and the sheer number of interacting variables that influence the outcome of each immune response. Ultimately, however, the state of PBV research and the sizeable impact that even case-by-case breakthroughs have on the veterinary and medical worlds more than justify continued research into structural vaccinology and the design of modified PBVs.

## Author Contributions

KS and FG initiated the review. All authors contributed to the writing and revision of the manuscript.

### Conflict of Interest

FG is employed by Locus Biosciences. TL is employed by GlaxoSmithKline. The remaining authors declare that the research was conducted in the absence of any commercial or financial relationships that could be construed as a potential conflict of interest.
